# Assessing Satisfaction in Simulation Among Medical Students: Psychometric Validation of the Italian Version of the Satisfaction with Simulation Experience Scale

**DOI:** 10.3390/nursrep16070235

**Published:** 2026-07-07

**Authors:** Samuel Agostino, Elena Casabona, Massimiliano Abate Daga, Franco Veglio, Alberto Milan, Grazia Papotti, Beatrice Albanesi

**Affiliations:** 1 Department of Medical Sciences, University of Turin, 10126 Turin, Italy; elena.casabona@unito.it (E.C.); massimiliano.abatedaga@unito.it (M.A.D.); franco.veglio@unito.it (F.V.); grazia.papotti@unito.it (G.P.); 2 Advanced Medical Simulation Center (SimTO), School of Medicine, University of Turin, 10126 Turin, Italy; 3 Candiolo Cancer Institute, Scientific Hospital and Treatment Center, 10060 Candiolo, Italy; alberto.milan@unito.it; 4 Department of Public Health and Pediatrics, University of Turin, 10124 Turin, Italy; beatrice.albanesi@unito.it

**Keywords:** simulation-based education, medical education, psychometric validation, satisfaction with simulation

## Abstract

**Background:** Simulation-based education (SBE) is increasingly integrated into medical curricula to enhance clinical reasoning, reflective practice, and experiential learning in safe educational environments. Evaluating learners’ satisfaction is essential to assess the educational impact of simulation activities. Although the Satisfaction with Simulation Experience Scale (SSE) has been internationally validated, no robust psychometric validation has been conducted among Italian medical students. This study aimed to evaluate the psychometric properties of the Italian version of the SSE (SSE-It). **Methods:** A cross-sectional psychometric validation study was conducted with 408 third-year medical students enrolled at a Northern Italian university after participation in a mandatory simulation internship on introductory ultrasound. Participants completed the Italian adaptation of the 18-item SSE, rated on a 5-point Likert scale and organized into three domains: Debriefing and Reflection, Clinical Reasoning, and Clinical Learning. Internal consistency was assessed using Cronbach’s alpha and McDonald’s omega coefficients. Confirmatory factor analysis (CFA) was performed to examine the factorial structure of the scale. **Results:** CFA supported the original three-factor structure of the SSE-ITA, showing good fit indices: χ^2^(132) = 410.67 (WLSMV-scaled), CFI = 0.98, TLI = 0.97, RMSEA = 0.07, and SRMR = 0.05. Internal consistency was high for the total scale (α = 0.94; ω = 0.95) and satisfactory across subscales (α ranging from 0.84 to 0.92; ω ranging from 0.86 to 0.93). **Conclusions:** The SSE-It demonstrated satisfactory reliability and construct validity in this task-trainer-based, introductory ultrasound simulation context, supporting its use as a reliable instrument to assess satisfaction with this type of simulation-based education among Italian medical students. Generalizability to high-fidelity, immersive simulation modalities remains to be established.

## 1. Introduction

Education in healthcare has undergone a substantial transformation with the integration of clinical simulation into undergraduate curricula. Indeed, simulation-based education or learning (SBL) allows students to practice in safe and controlled environments, fostering the development of both technical and non-technical skills [1].

Evidence from systematic reviews and recent empirical studies suggests that simulation improves knowledge and skill acquisition, supports knowledge retention, enhances clinical reasoning and decision-making, and increases learners’ self-confidence and satisfaction more effectively than traditional teaching methods [2]. SBL is also increasingly recognized for developing collaborative and interprofessional competencies among healthcare students. A recent systematic review found that SBL activities enhance team communication and interprofessional interaction, essential for safe and effective patient care [3,4].

Evaluating students’ satisfaction within SBL is a key component in determining the effectiveness of educational interventions. Valid and reliable instruments are essential for collecting meaningful feedback and supporting the continuous improvement of SBL. Recent research has highlighted the importance of assessing learner satisfaction and self-confidence as meaningful indicators in SBL, given their documented association with student engagement and perceived learning outcomes [3,5]. These dimensions not only reflect learners’ experiences but also contribute to understanding how simulation influences motivation, preparedness for clinical practice, and overall educational quality.

Despite the widespread integration of simulation into healthcare education, there remains a limited availability of psychometrically validated instruments—particularly within the Italian context—to assess students’ satisfaction with simulation experiences [6]. Cross-culturally adapted and validated tools are essential to ensure conceptual equivalence, relevance, and applicability across different educational and cultural settings [7]. Developing and validating such measures represents a fundamental step for strengthening evaluation practices and advancing high-quality SBL.

## 2. Background

Simulation-based learning encompasses a range of educational modalities designed to replicate clinical environments without exposing real patients to risk, including high-fidelity manikin-based simulation, task trainers, standardised patients, and virtual or augmented reality platforms [1,8]. Each modality offers distinct affordances for skill development, and evidence suggests that student satisfaction may vary accordingly, with immersive formats generally associated with higher perceived educational value, though findings remain heterogeneous [5]. Simulation-based learning (SBL) is increasingly recognized as a core component of health professions education, particularly in medicine or nursing, where it facilitates the development of clinical reasoning, decision-making, and technical skills in a safe and controlled environment [5]. SBL allows students to face complex clinical scenarios that may not be readily available during traditional clinical placements, offering repeated exposure, structured feedback, and opportunities for reflection [7,8]. These experiences are particularly valuable in an era in which access to clinical environments is often limited due to patient safety concerns, legal restrictions, and increased demand for placement sites [9]. Moreover, as healthcare systems evolve and clinical placements become more constrained, simulation provides a valuable supplement to experiential learning [10,11]. Beyond technical preparation, simulation supports the integration of cognitive, affective, and behavioral competencies. Simulated scenarios can be designed to address teamwork, communication, ethical dilemmas, and urgent clinical decision-making, often in high-stakes environments such as emergency care, maternal-child health, or intensive care settings [12,13]. These dimensions of learning are increasingly emphasized in competency-based nursing education and reflected in international guidelines [14,15], which recommend the use of simulation to promote clinical judgment, adaptability, and readiness for practice in healthcare students.

In this context, student satisfaction has emerged as a fundamental metric for evaluating the quality and effectiveness of SBL. Satisfaction is a multidimensional construct that reflects learners’ perceptions of realism, educational value, and applicability to real-world clinical practice [16]. Studies indicate that high levels of satisfaction are linked to greater engagement, improved confidence, and a more positive attitude toward future clinical experiences [17,18]. In particular, students report that simulation helps consolidate classroom knowledge, enhances their self-awareness, and fosters a deeper understanding of their professional role [11,16]. Moreover, satisfaction has been shown to correlate with learners’ perceptions of the quality of debriefing, the opportunity to reflect, and the perceived transferability of learning to clinical practice [19]. As highlighted by Williams and Dousek, measuring satisfaction is not only helpful in evaluating specific simulation activities but also plays a key role in informing instructional design and ensuring the alignment of learning objectives with student needs [11].

To assess satisfaction in a standardized and validated manner, Levett-Jones et al. developed the Satisfaction with Simulation Experience (SSE) Scale, a psychometric instrument specifically designed to evaluate simulation experiences in undergraduate nursing education. The SSE captures three domains: (1) Debriefing and Reflection, (2) Clinical Reasoning, and (3) Clinical Learning. In a previous study, the SSE has demonstrated acceptable internal consistency (Cronbach’s α = 0.77) and construct validity through factor analysis and has been adopted in various educational contexts internationally. The Australian validation study by Williams and Dousek (2012) [11] confirmed the three-factor structure of the SSE among paramedic students and reported a Cronbach’s α of 0.88 for the overall scale. Similarly, Slovakian and Croatian adaptations [20,21] of the SSE have reported robust psychometric properties, supporting the instrument’s reliability and cultural adaptability.

In the Italian educational context, where SBL is increasingly integrated into both nursing and medical curricula, few validated tools are available to systematically assess learners’ satisfaction. The first Italian adaptation of the Satisfaction with Simulation Experience Scale (SSE-ITA) was conducted by Guasconi et al. (2021) [22], who examined face and content validity and reported satisfactory reliability in a small sample of nursing students. More recently, Alberti et al. (2024) [23] carried out a more advanced psychometric evaluation of the SSE among undergraduate nursing students, confirming the original three-factor structure and demonstrating excellent internal consistency (Cronbach’s α = 0.94). However, to the best of our knowledge, no evidence is currently available regarding the validity of the SSE in medical education. Extending validation to different learner populations strengthens the robustness of an assessment tool, ensuring that its dimensional structure, reliability, and interpretability are preserved when applied in new educational settings [24]. Therefore, the present study aims to address this gap by examining the structural validity, internal consistency, and construct validity of the Italian version of the SSE among third-year medical students. By doing so, it expands the applicability of the SSE beyond nursing contexts and provides medical educators and researchers with a reliable instrument for evaluating learners’ satisfaction, supporting quality assurance processes, and informing curriculum development. This extension is directly relevant to nursing science and nursing education: because the SSE was originally developed and validated within nursing curricula, and is increasingly used across the interprofessional simulation activities that bring nursing and medical students together, establishing its psychometric equivalence in a medical student cohort allows nurse educators and researchers to use the scale as a shared, validated benchmark for satisfaction across professional groups, rather than relying on profession-specific tools whose comparability has not been established. In Italy, an initial validation of the Satisfaction with Simulation Experience—Italian Version (SSE-ITA) was conducted with 10 undergraduate nursing students and included an assessment of content validity [22]. However, as the authors noted, a larger sample was required to confirm the psychometric robustness of the newly validated instrument. In addition, the research team recommended evaluating the scale across different settings and student cohorts to generate further evidence of reliability and construct validity [22].

## 3. Materials and Methods

### 3.1. Design Setting and Participants

A cross-sectional validation design was used [22,25]. All 455 third-year medical students enrolled in the Faculty of Medicine at the University of Turin who attended the mandatory simulation internship accessed the questionnaire and provided informed consent. Of these, 47 did not complete the SSE item set (all-or-none missingness, consistent with survey abandonment rather than item-level non-response) and were excluded from all psychometric analyses, yielding an analytic sample of n = 408, which was also used for the test–retest reliability analysis. The adequacy of the sample size was established according to the item/participant ratio of a maximum of 1:10 [26], yielding a minimum required sample of 180 participants for the 18-item SSE. For confirmatory factor analysis, a minimum of 200 participants is generally recommended to obtain stable parameter estimates.

### 3.2. Data Collection

Data were collected between March and May 2024 using an anonymous online questionnaire distributed through Google Forms [27], administered at the end of a mandatory simulation internship titled “Introduction to Ultrasound” held annually at SimTO. The internship consisted of a single half-day session (4 h), structured as a brief theoretical introduction followed by hands-on practice across four rotating stations (30 min each), where clinical cases were simulated using task trainers under the guidance of trained clinical tutors. A structured debriefing was conducted at the end of each session. While third-year students had prior exposure to simulation-based learning during their second year (focused on clinical examination skills), none had previously received practical training in ultrasound; the internship therefore represented their first hands-on experience with this specific modality. The survey required approximately 15 min to complete. The questionnaire was completed by students immediately following the simulation session, prior to leaving the simulation centre.

### 3.3. Instrument Description

The questionnaire comprised three sections: (i) sociodemographic data (age, gender, previous simulation experience); (ii) educational information (internship completion, exposure to simulation activities); and (iii) the Italian version of the SSE.

The SSE is an 18-item self-report instrument developed by Levett-Jones et al. (2011) [19] to assess students’ satisfaction with simulation-based education, rated on a 5-point Likert scale (1 = Strongly Disagree to 5 = Strongly Agree). Items are organized into three factors: Debriefing and Reflection (Q1–Q9), capturing the perceived quality of facilitator feedback and reflective learning; Clinical Reasoning (Q10–Q14), measuring the enhancement of decision-making and clinical reasoning skills; and Clinical Learning (Q15–Q18), reflecting perceived skill development and integration of theoretical knowledge into practice. Factor scores are calculated as the mean of the corresponding items; a total score is obtained by summing all 18 items, with higher scores indicating greater satisfaction.

### 3.4. Data Analysis

Psychometric testing followed established guidelines for health measurement instrument validation [25] and proceeded in two phases.

In the first phase, descriptive statistics (mean, SD, frequencies, percentages) were computed for sociodemographic variables and item responses; skewness and kurtosis within ±1 indicated normal distribution [28].

In the second phase, factorial validity was examined via confirmatory factor analysis (CFA) on complete cases (n = 408). Given the all-or-none missingness pattern on SSE items, listwise deletion was adopted rather than imputation, consistent with the complete-data requirements of the WLSMV estimator and EGA network estimation. Preliminary factorability was assessed using Bartlett’s sphericity test and the Kaiser-Meyer-Olkin (KMO) index. Given the ordinal nature of the 5-point Likert items, CFA was estimated using the weighted least squares mean- and variance-adjusted (WLSMV) estimator. The original three-factor structure of the SSE [19,21] was compared against an alternative two-factor solution (with Clinical Reasoning and Clinical Learning merged into a single factor) using the scaled chi-square difference test. Fit was evaluated using CFI/TLI (≥0.90 acceptable), RMSEA (≤0.08 acceptable) [29], and SRMR (≤0.08 acceptable) [30,31]. Internal consistency was assessed via Cronbach’s α and McDonald’s ω (≥0.70 acceptable). An Exploratory Graph Analysis (EGA) complemented the CFA post hoc; as it was performed on the same sample, its results were interpreted with caution.

Test–retest reliability was assessed in the same 408 participants. Reliability was quantified using intraclass correlation coefficients (ICC) under a two-way mixed effects model, estimating both absolute agreement [ICC(A,1)] and consistency [ICC(C,1)]. The standard error of measurement (SEM) and minimal detectable change at 95% confidence (MDC_95_) were computed as SEM = SD_pooled × √(1 − ICC) and MDC_95_ = SEM × 1.96 × √2. No missing values were observed on SSE items; missing data were limited to dominant hand (n = 5, 1.2%) and did not affect psychometric analyses. All analyses were conducted using IBM SPSS Statistics 26.0, JASP 0.19.3.1, and R 4.6.0.

### 3.5. Ethical Considerations

The original author of the SSE granted formal authorization for its use and cross-cultural validation. The Bioethical Committee of the University of Turin reviewed and approved the study protocol (No. 219864). Eligible students received both verbal and written information about the study’s objectives, procedures, and the voluntary nature of participation. All participants provided electronic informed consent before completing the questionnaire, and data were collected anonymously following data protection regulations.

## 4. Results

A total of 408 medical students participated in the study. Most were female (67.4%), with a median age of 22 years (IQR = 1). Most had attended the clinical methodology course (77.2%) or already passed its exam (19.4%), were right-handed (93.1%), and had no prior ultrasound experience (92.9%). Detailed sociodemographic and educational characteristics are presented in Table 1.

### 4.1. Psychometric Testing

The mean score of each item of the SSE ranged from 3.94 to 4.69. The lowest mean score was observed for Item 13 (“The simulation helped me to recognize patient deterioration early”; M = 3.94, sd= 0.99), while the highest mean score was found for Item 4 (“The debriefing provided an opportunity to ask questions”; M = 4.69, sd = 0.59), Item 14 (“This was a valuable learning experience”; M = 4.69, sd = 0.56), and Item 15 (“The simulation caused me to reflect on my clinical ability”; M = 4.69, sd = 0.62). Standard deviations ranged between 0.56 and 0.99, suggesting acceptable variability across responses. Table 2 provides detailed item-level descriptive statistics.

### 4.2. Dimensionality and Construct Validity

Bartlett’s test of sphericity was highly significant (χ^2^ = 4862.25, df = 153, *p* < 0.001), and the Kaiser-Meyer-Olkin (KMO) index showed excellent adequacy (overall KMO = 0.95; all item MSA values ≥ 0.93), confirming the dataset’s suitability for factor analysis. Given the ordinal nature of the 5-point Likert items, confirmatory factor analysis (CFA) was estimated using the WLSMV estimator on complete cases (n = 408) to test the original three-factor structure of the SSE-IT. The model showed good fit: χ^2^ (132) = 410.67 (scaled), *p* < 0.001; CFI = 0.98; TLI = 0.97; RMSEA = 0.07 (90% CI: 0.064–0.080); SRMR = 0.05. An alternative two-factor model, merging Clinical Reasoning and Clinical Learning into a single factor, also showed acceptable fit (CFI = 0.98; TLI = 0.97; RMSEA = 0.07; SRMR = 0.05); however, the scaled chi-square difference test favored the three-factor solution (Δχ^2^(2) = 17.90, *p* < 0.001), supporting retention of the original structure. These results are consistent with previous validations of the SSE in other cultural contexts, supporting the presence of three latent factors: Debriefing and Reflection, Clinical Reasoning, and Clinical Learning.

### 4.3. Internal Consistency and Reliability

Internal consistency estimates for the SSE-IT were high across all factors (Table 3). The total scale showed excellent reliability (Cronbach’s α = 0.94; McDonald’s ω = 0.95). Each subscale also demonstrated strong internal consistency: Debriefing and Reflection (α = 0.92, ω = 0.93), Clinical Reasoning (α = 0.84, ω = 0.86), and Clinical Learning (α = 0.86, ω = 0.86).

Inter-factor correlations were positive and statistically significant. The correlation between Clinical Reasoning and Clinical Learning was notably high (r = 0.92, *p* < 0.001), consistent with findings from other cultural adaptations of the SSE [21,32]. While this overlap may reflect the inherently intertwined nature of these constructs in simulation-based learning, it also raises questions about their empirical distinctiveness in this population. As reported in Section 4.2, a formal comparison between the three-factor model and an alternative two-factor solution (merging Clinical Reasoning and Clinical Learning) favored retention of the three-factor structure (Δχ^2^(2) = 17.90, *p* < 0.001), although the practical improvement in fit was modest (ΔCFI = 0.002), indicating that the two constructs, while statistically distinguishable, remain closely related and warrant further investigation in larger, multi-institutional samples.

### 4.4. Test–Retest Reliability

Test–retest reliability was assessed in the 408 participants. Total score ICC(A,1) = 0.918 (95% CI [0.590, 0.970]), classified as excellent, and ICC(C,1) = 0.955 (95% CI [0.950, 0.960]). At the subscale level, ICC(A,1) ranged from 0.847 (Clinical Learning) to 0.889 (Debriefing and Reflection), all classified as good (see Table S1). Item-level ICC(A,1) values ranged from 0.608 (Q6) to 0.812 (Q13), all in the moderate-to-good range (Table 4). SEM was 2.43 points and MDC_95_ = 6.74 points. A small but statistically significant practice effect was observed (mean T1−T2 difference = 2.42 points; Cohen’s d = 0.95), consistent across all subscales and not affecting ICC estimates.

### 4.5. Exploratory Graph Analysis

**Figure 1:**
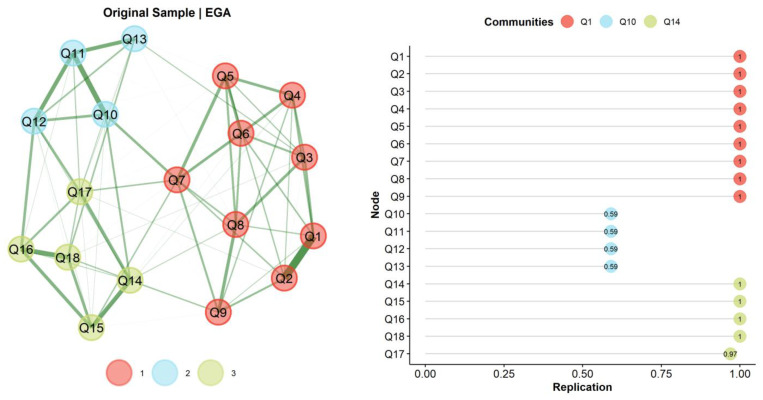
Exploratory Graph Analysis (EGA) network and item stability of the SSE-IT (N = 408).

An Exploratory Graph Analysis (EGA) (Figure 1) was conducted as a complementary, post hoc approach to explore the dimensional structure of the SSE-IT (n = 408). The network identified a three-community structure broadly consistent with the theoretical domains: Debriefing items (Q1–Q9) were fully stable (item stability = 1.00), while Item 14 clustered with Clinical Learning rather than Clinical Reasoning, and this boundary showed marginal stability (item stability = 0.58; three-dimensional solution replicated in 57.8% of bootstraps)—consistent with the high latent correlation between these factors observed in the CFA. As an exploratory, post hoc analysis, these results are reported for completeness alongside the confirmatory findings.

## 5. Discussion

This study validated the Italian version of the Satisfaction with Simulation Experience Scale (SSE-IT) in a large sample of undergraduate medical students, confirming the original three-factor structure—Debriefing and Reflection, Clinical Reasoning, and Clinical Learning—proposed by Levett-Jones. Consistent with established recommendations for psychometric validation [24], the analysis demonstrated solid evidence of structural validity through confirmatory factor analysis. The CFA results showed good fit indices (CFI = 0.98, TLI = 0.97, RMSEA = 0.07, SRMR = 0.05), meeting common criteria for model adequacy in applied research [29]. The scale also demonstrated excellent internal consistency (α = 0.94; ω = 0.95), supporting the reliability of scores across items and latent constructs. Evidence from systematic reviews confirms that simulation-based learning enhances clinical reasoning and critical thinking by exposing students to complex, realistic scenarios that require active problem-solving and decision-making [2,5]. The high scores observed on the Clinical Reasoning subscale are consistent with this evidence.

High mean item scores indicated overall positive perceptions of the simulation experience, with particularly strong ratings for items concerning opportunities to ask questions during debriefing, the perceived educational value of the session, and reflection on clinical abilities. The lowest-scoring item, related to recognizing early patient deterioration, may reflect limited exposure to acute-care scenarios within the specific simulation curriculum. The prior simulation experience of participants—who had attended at least one simulation-based session during their second year but had no previous exposure to ultrasound—may have contributed to the high satisfaction scores observed. Familiarity with the simulation format likely reduced anxiety and facilitated engagement, while the novelty of ultrasound as a specific modality may have heightened perceived educational value. The rotating station structure, combining hands-on practice with structured debriefing, is consistent with instructional designs known to promote active learning and reflective practice [1,5]. These findings position the SSE-IT as a valid and reliable instrument for evaluating medical students’ satisfaction with simulation-based learning and contribute to filling an important gap in the Italian educational context. The results are consistent with previous international validations of the SSE, including Croatian and Korean studies that similarly reported a stable three-factor structure and high reliability [21,32]. Compared with the preliminary Italian adaptation by Guasconi et al. (2021) [22], which focused on face and content validity in a small nursing sample, the present study provides a more rigorous and comprehensive psychometric evaluation, in line with best-practice guidelines for scale validation that emphasize the need to assess dimensionality, internal consistency, and construct validity in multiple populations [33].

High levels of satisfaction found in this study reflect broader evidence linking satisfaction and self-confidence with student engagement, motivation, and perceived learning outcomes in simulation-based education [34]. Satisfaction may also vary depending on the simulation modality employed. High-fidelity and immersive formats have been associated with greater perceived educational value compared to lower-fidelity approaches, though findings across studies remain heterogeneous [5,18]. In the present study, the use of task trainers in a rotating station format may have contributed to the uniformly high satisfaction scores by providing repeated, structured exposure to the same clinical skills [35]. Nevertheless, these high scores may partially reflect the novelty effect associated with simulation, especially in contexts where simulation is still being consolidated within the curriculum. Longitudinal studies could clarify whether satisfaction remains stable as students gain more frequent and diversified exposure to simulation activities.

The role of the facilitator is a critical determinant of simulation quality and learner satisfaction. Facilitator competence—including the ability to structure debriefing, balance guidance with autonomy, and provide constructive feedback—has been shown to directly influence how students perceive the educational value of simulation [19,34]. Debriefing has been consistently identified as one of the most influential components of simulation-based learning, with structured approaches such as PEARLS or advocacy-inquiry associated with deeper reflection and higher learner satisfaction [19]. The strong ratings observed on Debriefing and Reflection items in this study suggest that facilitator performance at SimTO was perceived positively by participants, and that the structured debriefing format adopted contributed meaningfully to overall satisfaction.

Although the updated CFA showed good model fit overall, cross-cultural nuances in how Italian medical students interpret specific items—shaped by local debriefing practices, curricular framing of clinical reasoning tasks, or institutional simulation infrastructure—should still be considered when comparing SSE scores across settings [1,8].

These findings carry direct implications for nursing education and practice. Because the SSE was originally developed and validated within nursing curricula, demonstrating its robustness in a cohort of medical students strengthens its value as a shared outcome measure for the interprofessional simulation activities that increasingly bring nursing and medical students together in contemporary health professions education. Nurse educators who coordinate or evaluate such interprofessional sessions can therefore draw on a single, psychometrically sound instrument to benchmark satisfaction across professional groups, rather than relying on profession-specific tools whose comparability has not been established. Moreover, because satisfaction is closely linked to engagement, self-confidence, and reflective learning, a validated Italian instrument such as the SSE-IT can support nursing faculties in monitoring and improving the quality of simulation-based curricula, informing debriefing practices, and guiding resource allocation for simulation centres shared across nursing and medical programmes.

### Limits and Strengths

Generalizability is limited by the single-institution design and the exclusive enrolment of third-year medical students in one specific simulation activity (introductory ultrasound); whether the SSE-IT’s psychometric properties hold across different curricula, modalities, or professions remains to be established. The cross-sectional design prevents evaluation of change over time, and voluntary participation may have introduced self-selection bias.

The test–retest interval (4–42 days) was too heterogeneous to be optimal: shorter intervals risk recall bias, while longer ones risk memory decay and genuine perceptual change. This may have introduced measurement heterogeneity not fully captured by the overall ICC; a systematic T1–T2 difference (mean difference = 2.42, Cohen’s d = 0.95) further suggests a practice effect. Future studies should adopt a narrower, standardised window (e.g., 7–14 days).

A further limitation concerns the discriminant validity between Clinical Reasoning and Clinical Learning, which showed a high latent correlation (r = 0.92). A competing two-factor model (merging these factors) was formally tested against the three-factor solution: although the three-factor model fit significantly better (scaled χ^2^ difference test, *p* < 0.001), the practical improvement was modest (ΔCFI = 0.002), indicating the two constructs remain closely related in this sample—consistent with other cultural adaptations of the SSE [21,32]. This may partly reflect participants’ stage of training: as third-year students in their first exposure to this simulation modality, they may not yet clearly differentiate exercising clinical reasoning from perceiving its educational value. This interpretation remains speculative and warrants testing in learners at different training stages. Additionally, the 47 participants excluded for incomplete SSE responses could not be compared with completers on demographic or outcome variables, as no usable data were available for this subgroup.

Finally, convergent and cross-cultural validity evidence is currently absent; the former could be assessed against related measures, as in Tüzer et al. [36], and the latter by pooling datasets across countries. Despite these limitations, findings are broadly consistent with prior validation work [22,23,37], supporting the scale’s overall structure while highlighting the Reasoning–Learning boundary as a priority for future refinement.

## 6. Conclusions

This study provides solid evidence for the validity and reliability of the SSE-IT among undergraduate medical students in the context of a task-trainer-based, introductory ultrasound simulation, confirming its three-factor structure and excellent internal consistency. The instrument offers nurse educators and medical educators alike a practical, cross-professionally validated tool for assessing satisfaction with this type of simulation-based learning and informing curricular improvement in the shared simulation settings that increasingly characterize nursing and medical training. As satisfaction profiles may differ across simulation modalities, further studies should establish whether these psychometric properties extend to high-fidelity, immersive contexts and to other clinical disciplines.

## Figures and Tables

**Table 1:** Sample Sociodemographic and Educational Characteristics (n = 408).

Variable	n (%)
Gender, female	275 (67.4)
Age, median (IQR)	22 (1)
*Clinical methodology status*	
Currently attending	14 (3.4)
Attended course	315 (77.2)
Passed exam	79 (19.4)
Dominant hand *	
Right	375 (93.1)
Left	28 (6.9)
*Previous ultrasound experience*	
No	379 (92.9)
Yes	29 (7.1)

Note. IQR = interquartile range. * Based on valid responses (n = 403; missing = 5).

**Table 2:** Item Descriptive Characteristics (N = 408).

Item	M (SD)	Skewness	Kurtosis
Item 1. The facilitator provided constructive criticism during the debriefing.	4.43 (0.77)	−1.13	0.57
Item 2. The facilitator summarized important issues during the debriefing.	4.55 (0.69)	−1.56	2.41
Item 3. I had the opportunity to reflect on and discuss my performance during the debriefing.	4.32 (0.89)	−1.32	1.53
Item 4. The debriefing provided an opportunity to ask questions.	4.69 (0.59)	−2.09	5.20
Item 5. The facilitator provided feedback that helped me develop clinical reasoning.	4.57 (0.69)	−1.70	3.00
Item 6. Reflecting and discussing improved my learning.	4.65 (0.61)	−1.85	4.11
Item 7. The facilitator’s questions helped me learn.	4.63 (0.63)	−1.80	3.51
Item 8. I received feedback that helped me learn.	4.54 (0.70)	−1.65	3.16
Item 9. The facilitators made me feel comfortable during the debriefing.	4.69 (0.60)	−1.89	3.91
Item 10. The simulation developed my clinical reasoning skills.	4.51 (0.65)	−1.15	0.58
Item 11. The simulation developed my decision-making ability.	4.25 (0.82)	−0.91	0.53
Item 12. The simulation enabled me to demonstrate my clinical reasoning.	4.38 (0.74)	−1.03	0.59
Item 13. The simulation helped me recognize patient deterioration early.	3.94 (0.99)	−0.66	−0.20
Item 14. This was a valuable learning experience.	4.69 (0.56)	−1.29	1.63
Item 15. The simulation caused me to reflect on my clinical ability.	4.57 (0.62)	−1.24	1.13
Item 16. The simulation tested my clinical ability.	4.48 (0.71)	−1.29	1.73
Item 17. The simulation helped me apply what I learned.	4.52 (0.64)	−1.15	0.96
Item 18. The simulation helped me recognize my clinical strengths and weaknesses.	4.43 (0.73)	−1.11	0.58

Note. SD = standard deviation.

**Table 3:** Internal Consistency and Latent Factor Correlations.

	Composite ω	Cronbach’s α	F1 Debriefing	F2 Reasoning	F3 Learning
F1 Debriefing	0.93	0.92	—	0.70 **	0.72 **
F2 Reasoning	0.86	0.84	0.70 **	—	0.92 **
F3 Learning	0.86	0.86	0.72 **	0.92 **	—
Total scale	0.95	0.94	—	—	—

Note. ω = McDonald’s omega; α = Cronbach’s alpha. Correlations represent standardized latent factor correlations extracted from the confirmatory factor analysis (CFA) model. ** *p* < 0.001.

**Table 4:** Item-Level Intraclass Correlation Coefficients—Test–Retest Reliability of the SSE-IT (N = 408).

*Item*	*ICC (A,1)*	*95% CI Lower*	*95% CI Upper*	*p*
Q1	0.691	0.630	0.740	<0.001
Q2	0.741	0.680	0.790	<0.001
Q3	0.786	0.740	0.820	<0.001
Q4	0.615	0.510	0.700	<0.001
Q5	0.710	0.650	0.760	<0.001
Q6	0.608	0.520	0.680	<0.001
Q7	0.726	0.670	0.770	<0.001
Q8	0.743	0.690	0.790	<0.001
Q9	0.615	0.510	0.700	<0.001
Q10	0.710	0.640	0.760	<0.001
Q11	0.777	0.730	0.820	<0.001
Q12	0.703	0.640	0.750	<0.001
Q13	0.812	0.780	0.840	<0.001
Q14	0.645	0.560	0.710	<0.001
Q15	0.666	0.610	0.720	<0.001
Q16	0.752	0.700	0.790	<0.001
Q17	0.659	0.590	0.720	<0.001
Q18	0.742	0.690	0.790	<0.001

Note. ICC(A,1) = intraclass correlation coefficient, two-way mixed effects model, absolute agreement, single measures. 95% CI = 95% confidence interval. All *p*-values < 0.001. Item-level ICC values are expected to be lower than the total-score ICC due to reduced score variance at the single-item level. Interpretation thresholds: Moderate = 0.50–0.75; Good = 0.75–0.90; Excellent > 0.90.

## Data Availability

The data supporting the findings of this study are not publicly available due to privacy and ethical restrictions. However, anonymized data may be made available by the corresponding author upon reasonable request and subject to approval by an appropriate ethics committee, in accordance with institutional and regulatory requirements.

## Supplementary Materials

The following supporting information can be downloaded at: https://www.mdpi.com/article/10.3390/nursrep16070235/s1, Table S1: Subscale-Level Intraclass Correlation Coefficients—Test-Retest Reliability by SSE-IT Domain (N = 408). Reports the subscale-level test–retest reliability results for the SSE-IT domains. The table includes T1 and T2 mean scores and standard deviations, ICC(A,1), ICC(C,1), and 95% confidence intervals for each domain.

## Public Involvement Statement

No public involvement in any aspect of this research.

## Guidelines and Standards Statement

This manuscript was drafted in accordance with the COSMIN (Consensus-Based Standards for the Selection of Health Measurement Instruments) checklist for studies on the psychometric properties of patient-reported outcome measures, and with the STROBE (Strengthening the Reporting of Observational Studies in Epidemiology) statement for the cross-sectional component of the study design.

## Abbreviations

The following abbreviations are used in this manuscript:

CFA	Confirmatory Factor Analysis
CFI	Comparative Fit Index
CI	Confidence Interval
EGA	Exploratory Graph Analysis
KMO	Kaiser-Meyer-Olkin
MSA	Measure of Sampling Adequacy
RMSEA	Root Mean Square Error of Approximation
SBE	Simulation-Based Education
SBL	Simulation-Based Learning
SD	Standard Deviation
SRMR	Standardized Root Mean Square Residual
SSE	Satisfaction with Simulation Experience Scale
SSE-IT	Satisfaction with Simulation Experience Scale—Italian Version
SSE-ITA	Satisfaction with Simulation Experience Scale—Italian Adaptation
TLI	Tucker–Lewis Index
WHO	World Health Organization
