# Supplementary material for: Assessing Satisfaction in Simulation Among Medical Students: Psychometric Validation of the Italian Version of the Satisfaction with Simulation Experience Scale
Source: Nurs Rep. 2026 Jul 7;16(7):235. doi: 10.3390/nursrep16070235 (PMC13414669; doi:10.3390/nursrep16070235)
Supplement: Supplementary file 1 [file nursrep-16-00235-s001.zip › nursrep-4361760-supplementary.pdf]

Table S1

Subscale-Level Intraclass Correlation Coefficients — Test-Retest Reliability by SSE-IT Domain (N = 408)

Domain	*T1 M (SD)*	*T2 M (SD)*	*ICC(A,1)*	*95% CI (A,1)*	*ICC(C,1)*	*95% CI (C,1)*
Debriefing and Reflection	41.07 (4.91)	39.78 (4.55)	0.889	[0.710, 0.940]	0.922	[0.910, 0.940]
Clinical Reasoning	21.77 (2.98)	21.10 (3.12)	0.885	[0.790, 0.930]	0.906	[0.890, 0.920]
Clinical Learning	18.01 (2.26)	17.54 (2.31)	0.847	[0.780, 0.890]	0.864	[0.840, 0.890]

Note. ICC(A,1) = intraclass correlation coefficient, two-way mixed effects model, absolute agreement, single measures. ICC(C,1) = two-way mixed effects model, consistency (excludes systematic bias). 95% CI = 95% confidence interval. Interpretation thresholds (Koo & Mae, 2016): Good = .75–.90; Excellent > .90. A statistically significant mean difference was observed between T1 and T2 across all subscales (all $p < .001$), suggesting a small practice effect that does not compromise ICC estimates.
